# Quantifying Fetal Reprogramming for Biomarker Development in the Era of High-Throughput Sequencing

**DOI:** 10.3390/genes12030329

**Published:** 2021-02-25

**Authors:** Fu-Sheng Chou, Krystel Newton, Pei-Shan Wang

**Affiliations:** 1 Division of Neonatology, Department of Pediatrics, Loma Linda University School of Medicine, Loma Linda, CA 92350, USA; KNewton@llu.edu; 2 PXT Research & Data Analytics, LLC, Rancho Cucamonga, CA 91739, USA; callie.pswang@zohomail.com

**Keywords:** preeclampsia, placental insufficiency, transcriptional biomarker

## Abstract

Gestational hypertensive disorders continue to threaten the well-being of pregnant women and their offspring. The only current definitive treatment for gestational hypertensive disorders is delivery of the fetus. The optimal timing of delivery remains controversial. Currently, the available clinical tools do not allow for assessment of fetal stress in its early stages. Placental insufficiency and fetal growth restriction secondary to gestational hypertensive disorders have been shown to have long-term impacts on offspring health even into their adulthood, becoming one of the major focuses of research in the field of developmental origins of health and disease. Fetal reprogramming was introduced to describe the long-lasting effects of the toxic intrauterine environment on the growing fetus. With the advent of high-throughput sequencing, there have been major advances in research attempting to quantify fetal reprogramming. Moreover, genes that are found to be differentially expressed as a result of fetal reprogramming show promise in the development of transcriptional biomarkers for clinical use in detecting fetal response to placental insufficiency. In this review, we will review key pathophysiology in the development of placental insufficiency, existing literature on high-throughput sequencing in the study of fetal reprogramming, and considerations regarding research design from our own experience.

## 1. Introduction

Despite progress in global efforts to reduce pregnancy-related maternal and neonatal mortality, pregnancy-related vascular disorders continue to prevail and negatively impact maternal–fetal health [1]. Accounting for 2–8% of pregnancies, pregnancy-related vascular disorders are responsible for 40% of preterm births largely due to medically indicated induction of labor being the only definitive treatment for this devastating condition [1]. It is generally accepted that abnormal placental vasculogenesis and angiogenesis are responsible for the development of increased vascular resistance, resulting in a spectrum of vascular disorders of pregnancy ranging from asymptomatic gestational hypertension to life-threatening eclampsia. The interruption of the highly regulated signaling network in placental development disrupts the interface between the mother and fetus resulting in an insufficient supply of oxygen and nutrients to the growing fetus, a condition called placental insufficiency.

In normal pregnancy, tissue and vascular remodeling mediated by local immune regulation takes place in the developing chorionic villi via trophoblast proliferation, differentiation, and invasion [2,3]. Myometrial spiral artery remodeling occurs during the second trimester, with relaxation of the high-resistant coiled vessels promoting blood flow to the maternal–fetal interface in the villi, allowing proper gas exchange and nutrient delivery. Further neovascularization and local tissue remodeling result in increased blood flow to the region, creating a favorable system to allow rapid fetal growth in the third trimester. When this process is interrupted, increased resistance persists in the placental vasculature, causing maternal hypertension and end-organ damage. Moreover, inadequate blood supply or placental insufficiency, leads to inadequate oxygen and nutrient delivery to the growing fetus and causes fetal growth restriction (FGR).

FGR is associated with a myriad of short- and long-term complications, with cardiometabolic derangement and neurodevelopmental abnormalities being the most prominent and well characterized [4]. Specifically, FGR has been associated with early-onset type 2 diabetes mellitus (DM) and hypertensive disorders in the offspring as early as the second to the third decade of life [5]. Literature indicates that reduced muscle mass, abnormally high glucocorticoid axis activity, and underdevelopment of pancreatic islet cells are some of the well characterized mechanisms for the early onset of clinical insulin resistance as a result of FGR [5]. Additionally, altered stiffness of the arterial vessel wall, fetal heart remodeling, excessive renin-angiotensin axis activity, and decreased nephron number are among the most well-established causes of early-onset hypertension [6,7,8]. FGR is also associated with neurodevelopmental delay, poor cognitive functioning, learning disability, as well as mood disorders [9,10,11,12]. It has been further demonstrated that growth-restricted infants, born at an extremely low gestational age, have the worse neurodevelopmental outcomes [13]. A paramount example being a recent elegant brain MRI study that illustrated FGR, rather than prematurity itself, is associated with delayed brain volume growth at 12-months corrected age [14]. Furthermore, there are multiple studies denoting that abnormal white matter development is associated with FGR [14,15,16].

## 2. Fetal Programming and Reprogramming

### 2.1. Fetal Developmental Program

Following fertilization, the human embryo follows a distinct pattern of growth and development to form the multiorgan body. Normal fetal programming occurs during the critical stages of tissue and organ development and has a long-term impact on health and disease [17,18]. Fetal programming is time-dependent and relies on epigenetic regulation to modulate gene expression during different stages of tissue development. Epigenetics refers to the molecular mechanisms regulating gene expression without changing the DNA sequence. Regulation of gene expression machinery assembly by DNA methylation and histone modifications are some of the best characterized epigenetic mechanisms in gene expression. From the developmental biology standpoint, the foundation of fetal programming lays in the evolution of histone modification and DNA methylation orchestrated to influence gene expression profiles in the instruction of progenitor cell differentiation into functional terminal cells [17,19,20]. The efficiency of the epigenetic evolution, and the resultant balance between proliferation and terminal differentiation during fetal development, are affected by the genetic predisposition of the individuals. Moreover, paracrine signaling from the embryo’s microenvironment, as well as hormonal signaling from the placenta, also play crucial roles in the regulation of fetal programming in influencing fetal growth and development.

### 2.2. Fetal Reprogramming

In the context of placental insufficiency, the signaling pathways instructing the evolution of gene expression profiles in proper fetal development is altered at least partly due to a state of chronic hypoxia and nutrient deprivation. Such fetal response to placental insufficiency, with resultant alteration in global expression, is viewed as “fetal reprogramming” [5,21]. In a sense, transcriptional regulation is “reprogrammed” in order for the body to cope with the environmental stress, with a goal of ensuring survival. At the molecular level, DNA methylation and histone modifications are two major mechanisms discovered to date to play crucial roles in transcriptional regulation, controlling access of the transcription machinery to the DNA to transcribe the genetic codes. As enzymes that are involved in DNA methylation and histone modifications require oxygen and other metals or organic cofactors for their function, fetal reprogramming is an inevitable consequence of placental insufficiency [22,23,24].

### 2.3. High-Throughput Sequencing Approach to Studying Fetal Reprogramming

Fetal programming and reprogramming have largely remained as a theory until the blossoming of the biotechnology industry after the human genome sequence was fully uncovered. Large-scale screening of differential gene expression was initially made possible using hybridization techniques in microarray assays [25]. This technique has been applied in studies regarding fetal programming during normal development as well as fetal reprogramming associated with FGR [26,27,28]. With the advent of high-throughput sequencing, the transcriptional evolution during fetal programming and reprogramming can be characterized with even better precision. High-throughput sequencing, also known as next-generation sequencing, replaced hybridization techniques used in microarray assays to directly sequence nucleic acids (DNA and RNA). Applications of high-throughput sequencing include DNA methylation analysis (i.e., unmethylated cytosine is chemically converted to uracil followed by sequencing, resulting in nucleotide C being read as T), RNA quantification (i.e., sequencing of complementary DNA synthesized based on RNA fragments extracted from biospecimens, followed by genome alignment and sophisticated statistical analysis to estimate RNA copy number for comparison of gene expression levels), and chromatin–protein interactions (i.e., sequencing of protein-bound chromatin fragments precipitated by protein-bound antibodies). High-throughput sequencing was first applied in animal models of placental insufficiency and FGR. For example, DNA hypermethylation of the beta cell Pdx1 gene was increased in a rat model of placental insufficiency, causing decreased Pdx1 gene expression levels; these changes were associated with insulin resistance and DM [29]. Similarly, a mouse model of protein deprivation found changes in DNA methylation in the promoter region of the angiotensin converting enzyme-1 gene, a key regulator of hypertension [30]. Detailed review on epigenetic responses to in utero insults and the developmental origins of health and disease is out of the scope of this review, but can be found in surplus in the literature [20,31].

In the past two years, several studies were published to report the use of high-throughput sequencing in characterizing fetal or placental reprogramming in response to gestational hypertensive disorders and/or placental insufficiency using human tissues (Table 1). Our group assessed transcriptomes of blood obtained at birth from 20 very preterm newborns (3 with preeclampsia, 6 with placental insufficiency, and 11 controls without any of the aforementioned diagnoses) [21]. In this pilot study, we characterized transcriptional regulation by conducting pairwise analyses among the three groups. We demonstrated that SLC25A42 was differentially expressed when comparing the placental insufficiency group to the preeclampsia and the control groups, but not when comparing the preeclampsia group to the control group. SLC25A42, a gene belonging to the soluble carrier family responsible for coenzyme-A and adenosine 3′,5′-diphosphate transport in human mitochondria, was thus found to be differentially upregulated in the placental insufficiency group.

Another study examined the changes in developmental programming profiles in the human umbilical vein endothelial cells following FGR [32]. Using RNA sequencing and transcriptome analysis, they identified four differentially expressed genes (downregulation of LGALS1, FPR3, and NRM; upregulation of RP5-855F14.1). Additionally, DNA methylation assays were also performed, which revealed hypomethylation of FPR3 and hypermethylation of NRM. The roles of these genes are not immediately related to tissue growth and metabolism upon literature search.

Ranzil et al. took a targeted approach by examining the placental serotonin synthesis pathway in response to FGR in the 10–12 week gestation human placentae, and found differential expression and activity of key components of the serotonin synthesis pathway associated with FGR pathogenesis [33].

Hannan et al. used RNA sequencing to identify circulating mRNAs that are differentially expressed in pregnancies with severe placental insufficiency and at high risk of stillbirth [34]. Five mRNAs (NR4A2, EMP1, PGM5, SKIL, and UGT2B11) were found to be differentially expressed in severe placental insufficiency. These genes either regulate cell proliferation (NR4A2, EMP1, and SKIL) or are involved in carbohydrate metabolism (PGM5). The function of UGT2B11 is unclear, but it is known to be expressed in multiple tissue types [35]. Other than UGT2B11 being downregulated, the other four genes were upregulated in FGR, possibly a compensatory mechanism to maintain growth. Among these five genes, three of them (NR4A2, EMP1, and PGM5) were validated in a second cohort. The expression levels of EMP1 and PGM5 were sufficient to predict FGR, with an area under the curve of 0.92. Additionally, NR4A2, EMP1, and RCBTB2 were associated with stillbirth. In this study, their focus was placental insufficiency, regardless of preceding pathophysiology. Only 49% (*n* = 63) of pregnancies with placental insufficiency in the discovery cohort had a diagnosis of preeclampsia.

MicroRNA is one type of noncoding RNA with a specific role in regulating mRNA stability and controlling protein translational efficiency from mRNA, and is therefore, in broad terms, also involved in epigenetic regulation of gene expression [36]. Expression levels of microRNA in normal development and in disease conditions may also be assessed using RNA sequencing. Awamleh et al. sequenced mRNA and microRNA extracted from placentae in pregnancies complicated by preeclampsia, FGR, and preeclampsia with FGR [37]. They identified six microRNAs and 22 mRNAs that were differentially expressed in the placentae of pregnancies with complications but not in control placentae. Moreover, they identified five microRNAs (miR-193b-5p, miR-193b-3p, miR-210-5p, miR-365a-3p, miR-365b-3p) that may be involved in the regulation of 16 genes that are involved in immune response, cell motility, communication, and proliferation in preeclampsia and/or preeclampsia with FGR groups [37]. The same group further validated the findings with RT-PCR, and used molecular biology techniques to confirm regulation of two gene targets, CSF1 and ITGAM, by miR-210-5p [38]. Both genes are involved in macrophage activation, and were found to only be differentially regulated in the group with preeclampsia and FGR, suggesting that a macrophage-related inflammatory pathway may be involved in placental insufficiency [39].

## 3. Considerations in the Use of High-Throughput Sequencing in Fetal Reprogramming Biomarker Development

In addition to mechanistic understanding, one major goal of studying fetal reprogramming is to develop transcriptional biomarkers for clinical use. Given that preeclampsia-induced placental insufficiency remains a diagnostic challenge as it relies on the emergence of nonspecific symptoms that vary from woman to woman, and that there are no reliable screening tests for placental insufficiency, especially near-term gestation when the risk of pregnancy complications such as fetal demise rises, it is reasonable to pursue additional biomarkers to assist in diagnosis [40]. Moreover, the current delivery guidance is largely based on maternal organ dysfunction, the development of placental insufficiency, and a worsening biophysical profile (a late sign of ongoing fetal distress) [41]. In other words, there is a lack of the assessment of fetal stress in its early stages to be taken into consideration during the clinical decision making process of whether or when to deliver the fetus. With evidence of fetal reprogramming and transcriptional dysregulation associated with placental insufficiency and FGR, transcriptional biomarker development would be a reasonable approach which would potentially allow early assessment of fetal response and adaptation to the toxic intrauterine environment. Several considerations are raised in the face of this approach and are discussed below.

### 3.1. Tissue Collection

The impact of placental insufficiency is global, as evidenced by multisystemic involvement of short- and long-term comorbidities associated with placental insufficiency and FGR [42,43]. However, the severity of insult and the degree of fetal reprogramming are likely a function of time and tissue type. Sex has also been shown to play a role in fetal response to FGR at the organismal level [44].

Rational development of biomarkers for fetal reprogramming requires thorough considerations of tissue types. While blood collected at time of birth may provide a comprehensive picture of the sum of antenatal stress and is reasonable for use in neonatal outcome prediction, it does not provide an opportunity for assessing the dynamics of fetal reprogramming, nor does it provide any value for clinical decision making in terms of optimal timing of delivery [21]. The placental or umbilical cord tissues are likely the tissue types that are the most sensitive to deprivation of oxygen and/or nutrients, and therefore provide an opportunity to assess reprogramming at its earliest stage [32,33,37]. However, these tissue types do not necessarily reflect the growth and development of the fetal body, and their assessment may not be feasible until after the fetus is delivered. Moreover, the samples that were obtained for research were only available after delivery of the fetus. Therefore, umbilical and placental tissues are not likely to serve as feasible biospecimen sources if the goal is to assess the response to fetal reprogramming antenatally. Moreover, blood and placental tissues are composed of various cell types from where RNA is extracted. As an example, it is well established that FGR is associated with hematological abnormalities in all three blood lineages [45,46]. Therefore, caution must be taken when interpreting gene expression comparison between the control and FGR groups. It remains to be determined whether it is necessary, and how, to normalize transcript counts to differences in cell type composition among samples.

Amniotic fluid biomarkers, in our opinion, are also not a feasible option for similar reasons. In addition, although all cell-free RNA in the amniotic fluid were derived from fetal tissues, the numbers of genes that were recovered seemed to exhibit intersample variability [28,47]. Due to ethical concerns, amniotic fluid collection before birth could only be done when a clinical test (usually for karyotyping or other genetic testing) that requires amniotic fluid collection is indicated, raising the concerns for generalizing the findings for mechanistic insights and/or diagnostic development [27,28,47].

On the other hand, assessment of circulating cell-free fetal RNA (cffRNA) seems to be a reasonable approach, as in theory, it allows assessment of the dynamic nature of fetal reprogramming in a longitudinal manner [34]. The cffRNA is shed from the placenta and the fetal organs into the maternal circulation [48,49]. Therefore, cffRNA may reflect transcriptome changes in the growing fetus when the specimen is collected at various time points as the pregnancy progresses. Nonetheless, extracted RNA from the maternal bloodstream is a mixture of placental and fetal RNA, as well as RNA from the microbes that reside in or invade the maternal body, making the normalization process a challenge [50].

### 3.2. Subjects

Different from inbred animal models, human subjects recruited for this type of translational study come from various genetic backgrounds. There is baseline noise of the gene expression levels by itself. Sex disparities in response to intrauterine stress may also play a role. More subjects may not solve the issue, but insufficient number of subjects makes it difficult to normalize for these demographic factors in the analysis, not to mention the assessment of interactions between demographic factors and baseline gene expression variations. If each gene is treated as an independent variable, the number of subjects needed to reach a certain threshold of power is likely very different for each gene. How the power analysis is conducted based on *a priori* information, as in traditional clinical trials, depends on what type of the statistical analysis will be performed.

### 3.3. Sequencing Depth

The sequencing depth, in lay terms, refers to the total number of nucleotide bases to be synthesized during the sequencing process [51]. Strictly speaking, sequencing “depth”, or coverage, refers to how many times a nucleotide is read, which then translates into how confident we are in assigning the sequence of that very nucleotide. In addition to the confidence in assignment, the “deeper” the sequencing process, the more likely the differentially expressed genes that are in low abundance will be detected. Ultimately, it is a balance between the computing power and the funding availability, as it takes more time to synthesize a higher number of nucleotides, costs more, and it also takes more computing resources and time to align or map when there are more sequences. In our recent study, we used a sequencing depth of 25 million per sample. Forty percent of genes received a zero normalized count across all 20 samples, suggesting that this depth is not quite sufficient to detect genes expressed in low abundance.

### 3.4. Alignment/Mapping

In our experience, the rate of successful alignment or mapping of the sequenced fragments to the genome with the outbred CD-1 mouse model was around 90% (or above), while the rate was 60–70% with RNA derived from human samples. Single nucleotide polymorphism may play a role, although it can be minimized by manipulating the algorithm for alignment. Alternatively, RNA from foreign microbes may consist of the remaining 30–40% of the fragments. In our opinion, there are at least three aspects that are worth considering from this observation: first, it implies that a much higher number of sequencing depth may be needed to overcome the inevitability of sequencing nonhuman fragments. Secondly, it may be worth investigating whether the fragments that could not be aligned or mapped to the human genome play a role in fetal reprogramming and disease severity, especially in the era of microbiota being the plausible origins of human diseases. Thirdly, it remains to be determined whether these fragments should really be discarded, or can/should be used in normalization given their abundance.

### 3.5. Count Normalization

Different approaches have been used to normalize raw RAN sequencing fragment counts. Reads per million mapped reads (RPM) normalize counts to total mapped reads, but do not take transcript length into consideration. Reads (or fragments) per kilobase per million mapped reads (RPKM or FPKM) not only normalize counts to total mapped reads but also take transcript length into consideration. The advantage of the latter normalization approach lies in the fact that longer RNA transcripts have a higher chance of being sequenced given more absolute entry points for sequencing. Transcript per million (TPM) is similar to RPKM/FPKM in that it takes both transcript length and sequencing depth into consideration. The difference is the sequence of normalization operations [52]. TPM can be interpreted as transcript frequency for a specific gene. None of the normalization methods stated above are perfect. Most importantly is that gene expression abundance, quantified by high-throughput sequencing (as well as by traditional polymerase chain reaction), is relative rather than absolute.

In our study, we used the DESeq2 package from R for differential gene analysis [53]. Total raw counts were used for normalization. In other words, normalization is not performed by sample; instead, it was by experiment. Therefore, the number of samples used in each experimental group for differential gene expression analysis affects the calculation of normalized counts.

## 4. Conclusions and Future Directions

With the availability of high-throughput sequencing, transcriptional biomarkers have been studied for their applicability in cancer diagnosis, especially malignancies that are difficult to diagnosis in their early stages [54,55,56]. Additionally, differentially expressed genes uncovered from high-throughput sequencing have recently been used, in combination with machine learning algorithms, in the development of predictive models for premature birth and asthma [49,57]. The use of high-throughput sequencing to quantify fetal reprogramming, as well as the use of differentially expressed genes for outcome prediction, is still in its infancy. We reviewed existing literature in this area of research, and shared our experience and thoughts on study design and potential technical challenges, with the hope that more research will be invested in this area to allow a better understanding of fetal reprogramming in response to placental insufficiency and other intrauterine environment. Indeed, not only is long-term health and disease of the offspring affected by placental insufficiency and FGR, studies have also found maternal distress, prepregnancy health, and other pregnancy complications to affect offspring well-being [58]. We believe that in the study of the dynamics of transcriptional regulation in fetal reprogramming, combining cffRNA and high-throughput sequencing serves as the best approach. We are confident that this method can be used for developing clinically applicable predictive models to guide therapeutic interventions. Such a goal may be achieved by combining candidate differentially expressed genes with clinical and demographic data, and by applying machine learning algorithms. Our understanding of fetal programming and reprogramming, and its clinical applicability, will only continue to advance with the availability of sophisticated molecular tools such as the high-throughput sequencing technology.

## Figures and Tables

**Table 1:** List of publications that report differentially expressed genes associated with placental insufficiency or fetal growth FGR (fetal growth restriction).

Author	Year	Tissue Origin	Subject Number	Findings
Chou et al.	2020	Neonatal blood collected at time of birth	20 (11 control; 3 preeclampsia; 6 placental insufficiency)	Upregulation of SLC25A42 in the placental insufficiency group. Significant number of downregulated genes in the placental insufficiency group in comparison to the control group.
Terstappen et al.	2020	Umbilical vein endothelial cells	19 (11 FGR; 8 control)	Downregulated gene sets in FGR: immune or inflammatory pathways, cell cycle pathways, cardiovascular health and function. Upregulated gene sets in FGR: kidney development. Downregulation of LGALS1, FPR3, NRM, and upregulation of lincRNA RP5-855F14.1 in FGR in RNA sequencing study. Hypomethylation of FPR3, hypermethylation of NRM in FGR in DNA methylation study.
Ranzil et al. *	2019	Third trimester placental tissue	65 (42 control; 23 FGR)	Downregulation of TPH2 and SLC6A4 in FGR. Upregulation of the 5-HT receptor in FGR.
Hannan et al.	2020	Maternal blood 2 h prior to delivery in FGR; 28–34 weeks gestation in control	170 (42 control; 128 preterm FGR)	NR4A2, EMP1, PGM5, SKIL, and UGT2B1 are differentially expressed in FGR. NR4A2 and RCBTB2 are dysregulated in fetal acidemia.
Awamleh et al.	2019	Placental tissue collected within 30 min of delivery	79 (21 control; 20 preeclampsia; 18 FGR; 20 preeclampsia+FGR)	Six microRNAs and 22 genes were identified to be differentially expressed in all three patient groups when compared to the control group) miR-193b-5p, miR-193b-3p, and miR-210-5p likely orchestrate to regulate APLN, CSF1, and TYRO3 in preeclampsia+FGR, while they each may individually regulate expressions of other genes.

* This study took a targeted approach to discovery of differentially expressed genes.

## Data Availability

Not applicable.
